# Sex hormones and oxidative stress biomarkers of male Wistar rats treated with *Moringa oleifera* seed fractions

**DOI:** 10.5935/1518-0557.20190047

**Published:** 2019

**Authors:** Olawale Olaleye Obembe

**Affiliations:** 1 Department of Physiology, College of Health Sciences, Osun State University, Osogbo, Nigeria

**Keywords:** *Moringa oleifera* seeds, sperm toxicity, testosterone, antioxidants

## Abstract

**Objective:**

Aqueous extract of *Moringa oleifera* seeds have been reported to exert male reproductive toxicity. To elucidate the spermatoxic constituents, methanol and hexane fractions of *Moringa* seeds were administered to male rats.

**Methods:**

Methanol or hexane fractions (50 and 100 mg/kg) of *Moringa* seeds were administered orally to male rats for six weeks, while a control group concurrently received the vehicle. Thereafter, sperm profiles were examined on a microscope while sex hormones and antioxidants levels were measured from serum samples.

**Results:**

The methanol fraction of *Moringa* seeds significantly decreased testosterone, luteinizing hormone, sperm motility, and sperm count of treated rats when compared with controls. The hexane fraction of *Moringa* seeds had no effect on sex hormones or sperm profiles. Both methanol and hexane fractions significantly increased superoxide dismutase and catalase levels, while malondialdehyde levels decreased significantly.

**Conclusion:**

The methanol fraction - but not the hexane fraction - of *Moringa oleifera* seeds induced male reproductive toxicity. By its turn, the hexane fraction had a higher antioxidant effect.

## INTRODUCTION

Male infertility and impaired fecundity is a growing global health concern. An estimated 8-12% of couples have experienced some form of infertility, with causative factors in about 40% of the cases being traced back exclusively to the male partner of the couple (Kumar & Singh, 2015). Male infertility commonly results from interference with testicular spermatogenesis stemmed from environmental, behavioral, or genetic factors (Ito *et al*., 2004). Recent studies have indicated that causative agents of this interference arise from environmental and dietary sources. Medicinal plants have been beneficial to mankind and have been used use as adjuvant or therapeutic agents in the treatment of various diseases. However, reports on the toxic effects of some medicinal plants on male reproductive physiology leading to infertility abound in the literature. The antimalarial and antibiotic properties of *Quassia amara* (Ajaiyeoba & Krebs, 2003), *Alstonia boonei* (Iyiola *et al.*, 2011; Ajayi *et al*., 2015), and *Bulchhozia coriacea* seeds (Mbata *et al*., 2009; Okoli *et al*., 2010), to name a few, have been reported alongside their antisteroidogenic and antifertility properties (Raji & Bolarinwa, 1997; Raji *et al.,* 2005; Obembe *et al.*, 2012).

*Moringa oleifera* seeds have been described as a natural product with documented therapeutic potential and controversial effects on male reproductive physiology. *Moringa oleifera* is a medicinal plant commonly cultivated in the tropics. It belongs to and is the most widely known species of the plant family Moringaceae. *Moringa oleifera* is a medium-sized tree of a height up to 10 meters with thick, soft, corky, deeply fissured bark. It has impressive medicinal uses and significant nutritional value. The leaves are reputed to have antidiabetic (Gupta *et al*., 2012), antioxidant (Sadek, 2014; Shaat *et al*., 2017), and antitumor (Sadek *et al*., 2017) properties.

Zade *et al.* (2013) suggested that the aqueous extract of *Moringa oleifera* seeds may improve male sexual behavior due to an observed increase in libido, sperm count, mounting frequency, intromission frequency, and ejaculation latency with reduction in mounting latency, intromission latency, and post ejaculatory interval. However, the aqueous seed extract at the same dose was reported in a separate study to exert male reproductive toxicity, as observed from its deleterious effect on sperm motility, sperm count, and testicular androgen (Obembe & Raji, 2018). To elucidate the spermatoxic constituents of *Moringa oleifera* seeds, the fresh seeds were fractionated based on polarity and their hexane and methanolic fractions obtained. The effect of these fractions on sperm profile, sex hormones, and serum antioxidant levels were examined.

## MATERIAL AND METHODS

### Extraction of *Moringa* seeds

Pods of *M. oleifera* seeds were collected from a plant located in an open field in Ibadan, Oyo State, Nigeria. The plant sample was identified and authenticated at the herbarium of the Federal Research Institute of Nigeria (FRIN), Ibadan, where a specimen was deposited and Voucher number FHI No 111249 assigned. The seedpods were broken to expose the clean white seed, which was then air-dried and pulverized into a white powder (1,060.69g). The powdered seed was dissolved in distilled water and then partitioned with hexane and methanol to successively yield hexane (HFMS, 53.01g, 0.05% percentage yield) and methanol (MFMS, 50.48g, 0.05% percentage yield) fractions. The obtained hexane and methanol fractions were refrigerated.

### Drug preparation

The hexane fraction of *Moringa* seeds is non-polar. Treatment was therefore administered using Tween 80 (Sigma-Aldrich, USA) as the vehicle.

### Animal grouping

All rats were housed in the Central Animal House of Osun State University, and were fed with standard rat pellets and clean water *ad libitum*. All procedures in this study were carried out in accordance with the Guide for Care and Use of Laboratory Animals (National Research Council (US) Committee, 2011) and approved by the Research Ethics Committee of the College of Health Sciences, Osun State University, Osogbo, Nigeria.

Twenty-five male Wistar rats (180-200 g) were used in the study. The rats were randomly assigned into five groups of five individuals each. Group 1 served as the control and received vehicle (Tween 80) only; Groups 2 and 3 were treated with methanol fractions (50 and 100 mg/kg respectively); and Groups 4 and 5 received hexane fractions (50 and 100 mg/kg) of *M. oleifera* seeds. Treatment was administered once daily for six weeks. The rats were then anaesthetized using sodium pentobarbital (30 mg/kg) and sacrificed by cervical dislocation. The sex and visceral organs were excised, cleared of adhering tissues and weighed. Sperm was collected from the caudal epididymis and analyzed on a microscope. Prior to sacrifice, serum was obtained for assay of sex hormones and oxidative stress biomarkers.

### Sperm profile

The caudal epididymis was excised and lacerated. The sperm obtained was categorized as belonging to one of three motility categories - progressive, non-progressive, or immotile, according to World Health Organization (2010) guidelines. Progressive forward motility was counted and scored to the nearest 10. The epididymis was immersed in 5 ml normal saline in a measuring cylinder and the volume of saline displaced was taken as the sperm volume. Sperm viability was assessed based on the improved one-step eosin-nigrosin staining technique. A fraction of each suspension of sperm samples was mixed with an equal volume of eosin-nigrosin stain. Air-dried smears were prepared on glass slides for each of the samples according to the procedure described by Björndahl *et al.* (2003). Normal live sperm cells exuded the eosin-nigrosin stain while dead sperm cells took up the stain. Sperm count was done under a microscope with the aid of an improved Neubauer hemocytometer. Counting was done in five Thoma chambers (Obembe *et al*., 2012).

### Hormonal assay

Serum levels of testosterone, luteinizing hormone, and follicle stimulating hormone were assayed using commercially available enzyme-linked immunosorbent assay (ELISA) kits. The kits were obtained from Calbiotech Inc. (California, USA) and contained the respective enzyme label, substrate reagent, and quality control sample. Quality control was carried out at the beginning and at the end of the assay in order to ascertain acceptability with respect to bias and within variations. The testosterone kit used had a sensitivity of 0.075ng/ml with intra- and inter-assay variations of 3.9 and 4.3%, respectively. The luteinizing hormone kit had a sensitivity of 0.12mIU/ml, with intra- and inter-assay variations of 7.6 and 10.83%. The follicle stimulating hormone kit had a sensitivity 0.353mIU/ml, with intra- and inter-assay variations of 5.6 and 6.4%.

### Biochemical assay

Spectrophotometric assays for measuring oxidative stress biomarkers were carried out on the obtained serum samples. Malondialdehyde (MDA) levels were as described by Mihara & Uchiyama (1978), superoxide dismutase (SOD) levels were in accordance with Sun & Zigman (1978), and catalase levels were as described by Aebi (1984).

### Histopathology

The testis and epididymis specimens excised from the rats were fixed in 10% paraformaldehyde. They were then washed in graded doses of ethanol to remove inherent water. Ethanol was washed off by immersion in xylene. Tissue slides embedded in paraffin wax were prepared and stained on Hematoxylin and Eosin. Stained slides were cleared in xylene before they were mounted on a microscope for histological examination. Images at 400x magnification were processed on a calibrated ToupView Image analysis software based on the photomicrographs taken with an AmScope camera fitted to an AccuScope microscope (Yang *et al*., 2006).

### Statistical analysis

Data were expressed as mean ± standard error of mean (SEM). Comparisons of mean values were made by one-way analysis of variance (ANOVA) on SPSS version 16 (SPSS Inc., Chicago USA). *p*<0.05 was considered significant.

## RESULTS

### Effects of *Moringa* seeds on organ weight

Methanol and hexane fractions of *M. oleifera* seeds at administered doses had no effect on the weight of sex or visceral organs of treated rats when compared with controls (Table 1). 

**Table 1:** Relative organ weights (%) of rats treated with *Moringa* seeds

	Control	50 mg/kg MFMS	100mg/kg MFMS	50 mg/kg HFMS	100mg/kg HFMS
Testis	0.57±0.05	0.60±0.02	0.57±0.02	0.56±0.04	0.59±0.01
Epididymis	0.27±0.01	0.28±0.01	0.29±0.01	0.24±0.01	0.28±0.01
Seminal vesicle	0.51±0.08	0.44±0.04	0.39±0.07	0.46±0.02	0.47±0.07
Prostate	0.13±0.02	0.14±0.01	0.12±0.01	0.14±0.01	0.12±0.01
Liver	3.08±0.08	3.02±0.20	3.12±0.10	3.03±0.12	2.83±0.17
Kidney	0.30±0.01	0.31±0.02	0.31±0.01	0.31±0.01	0.30±0.01
Heart	0.32±0.01	0.33±0.01	0.32±0.02	0.36±0.01	0.36±0.03
Spleen	0.30±0.02	0.39±0.03	0.31±0.02	0.33±0.05	0.40±0.05
Brain	0.75±0.03	0.76±0.02	0.77±0.06	0.77±0.02	0.77±0.03
Lungs	0.78±0.05	0.86±0.04	0.75±0.06	0.80±0.06	0.68±0.06

Values are Mean±SEM, n=5. Treatment had no effect on the weight of sex and visceral organs.

### Effects of Moringa seeds on sperm profile

Rats treated with methanol fraction of *M. oleifera* seeds (MFMS) had significantly lower sperm motility and sperm counts when compared with controls. No significant effect was observed on sperm volume, viability, or morphology. Hexane fraction of *M.* oleifera seeds (HFMS) had no effect on any of the recorded sperm parameters (Table 2)

**Table 2:** Sperm profile of rats treated with *Moringa* seeds

	Control	50 mg/kg MFMS	100mg/kg MFMS	50 mg/kg HFMS	100mg/kg HFMS
Sperm motility (%)	94.10±1.00	84.00±2.45*	76.00±2.45*	84.00±3.67	85.00±4.18
Sperm viability (%)	97.40±0.60	96.80±0.73	96.80±0.73	96.80±0.73	96.80±0.73
Sperm volume (ml)	5.18±0.02	5.18±0.02	5.18±0.02	5.18±0.02	5.18±0.02
Sperm count (million/ml)	146.20±2.2	129.00±4.63*	120.00±3.70*	130.20±6.58	131.60±7.08
% Abnormal morphology	11.08±0.26	10.61±2.43	10.58±2.34	9.60±2.12	10.32±2.26

Values are Mean±SEM, n=5.

* *p*<0.05 indicates significant difference from controls.

### Effects of Moringa seeds on serum antioxidants

Serum SOD and catalase were significantly increased in all rats treated with MFMS and HFMS when compared with controls, while MDA significantly decreased in the rats treated with MFMS and HFMS (Table 3). However, HFMS had significantly greater effects on these antioxidants than MFMS. Serum SOD and catalase were significantly higher in rats treated with HFMS than in rats treated with MFMS. MDA was significantly lower in rats treated with HFMS (50mg/kg) when compared with rats treated with MFMS (50mg/kg).

**Table 3:** Effects of *Moringa* seed fractions on oxidative stress biomarkers

	Control	50 mg/kg MFMS	100 mg/kg MFMS	50 mg/kg HFMS	100 mg/kg HFMS
SOD (iµ/ml)	2.46±0.15	5.50±0.13*	5.74±0.31*	7.04±0.21*†	7.50±0.23*‡
Catalase (iµ/ml)	25.86±1.52	127.70±1.21*	134.08±10.16*	178.20±7.21*†	203.76±3.09*‡
MDA (nmol/L)	28.48±2.36	2.38±0.16*	1.86±0.10*	1.78±0.04*†	1.72±0.04*

Values are Mean±SEM, n=5.

* *p*<0.001 indicates significant difference from controls,

† *p*<0.05 indicates significant difference from MFMS (50 mg/kg) and

‡ *p*<0.05 indicates significant difference from MFMS (100 mg/kg).

### Effects of Moringa seeds on sex hormones

**Figure 1:**
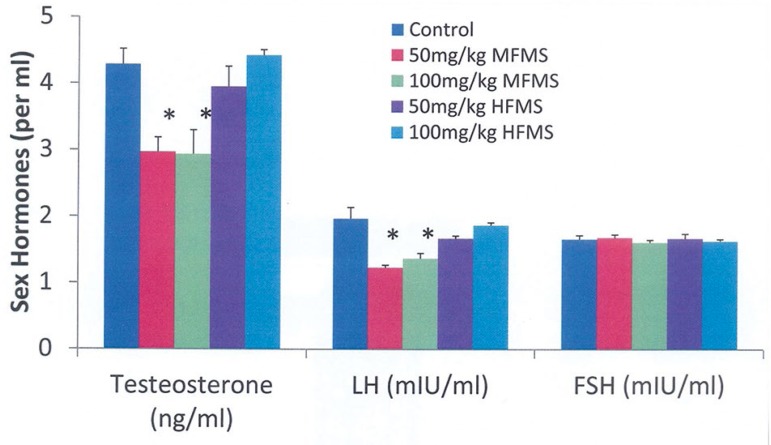
Sex hormones of rats treated with *Moringa* seed. Values are Mean±SEM, n=5. *p<0.05 indicates significant difference from controls. MFMS decreased serum testosterone and LH, while HFMS had no effect

Serum testosterone and LH were significantly reduced in MFMS-treated (50 and 100 mg/kg) rats, but no effect was observed on FSH. HFMS had no effect on testosterone, LH, or FSH (Figure 1).

### Histomorphology

**Figure 2:**
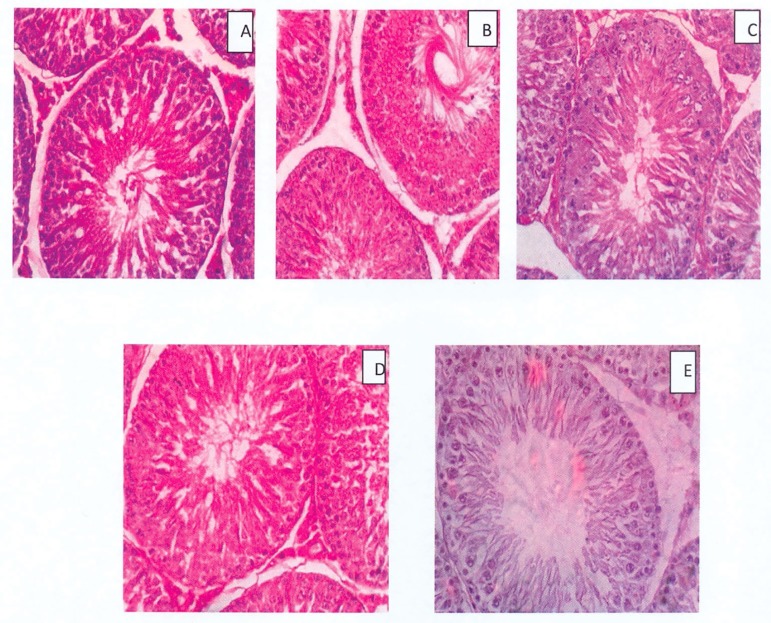
Histomorphology of the testes of rats treated with *Moringa* seeds (Mag. 400X). A- Control, B and C- 50 and 100 mg/kg MFMS, D and E- 50 and 100 mg/kg HFMS. No visible lesion was observed in any of the groups

**Figure 3:**
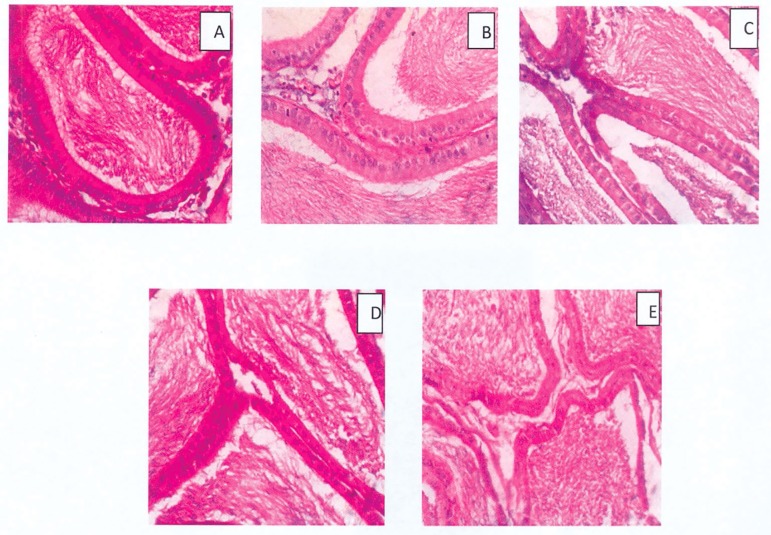
Histomorphology of the epididymis of rats treated with *Moringa* seeds (Mag. 400X). A- Control, B and C- 50 and 100 mg/kg MFMS, D and E- 50 and 100 mg/kg HFMS. No visible lesion was observed in any of the groups

Treatment with *Moringa oleifera* seeds had no significant effect on the histomorphology of rat testes when compared with controls (Figure 2). All groups had uniformly sized seminiferous tubules with regular outlines. The density of seminiferous tubules and spermatogenic cells of treated rats did not differ from controls. Additionally, *Moringa* seed treatment had no visible effect on the epididymis of treated rats when compared with controls (Figure 3). All groups had ducts that were lined with cuboidal epithelial cells with moderate to copious amounts of spermatozoa in the luminal spaces.

## DISCUSSION

The acceptability of medicinal plants for therapeutic purposes has continued to soar over the past decades. This is in view of their richness in bioactive principles and the perception that they are a desirable source of compounds for use in pharmaceutics and alternative medicine. However, unsolved reservations about many of these medicinal plants still persist in areas such as toxicity, dosage regimens, and effectiveness. In this study, *M. oleifera* seed extract was fractionated based on polarity, and the effect of the methanol and hexane fractions on sex hormones and serum antioxidants were examined. MFMS - but not HFMS - caused significant decline in testosterone and LH levels (Figure 1).

The observed decrease in testosterone may be due to the decrease seen in serum LH levels. Luteinizing hormone is produced by the gonadotropes of the anterior pituitary gland, and it stimulates the testicular Leydig cells to produce testosterone. A direct effect of MFMS on the testicular Leydig cells and seminiferous tubules cannot be ruled out, since sperm motility and sperm counts of samples obtained from the caudal epididymis were significantly decreased. Decreased serum testosterone levels may not account for the observed decline in sperm motility. Meeker *et al.* (2007) reported lack of a correlation between serum testosterone levels and sperm motility in infertile men. Serum testosterone levels were also described as having no correlation with sperm motility, concentration, pH, or morphology in buffalo bulls (Sajjad *et al*., 2007). The phytochemical constituents of MFMS probably mediated the outcomes on sex hormones and sperm profiles.

Documented phytochemical screening of *M. oleifera* seeds revealed the presence of alkaloids, saponins, tannins, terpenes, alkaloids, flavonoids, carbohydrates, and cardiac glycosides (Sinha, 2012; Ajibade *et al*., 2013; Idris *et al*., 2016). Some of these constituents - flavonoids and saponins, for example - have however been implicated in male reproductive toxicity. Rutin, a flavonoid commonly found in nature, has anti-mitotic, vasoprotective, and antihyperlipidemic activity. When hydrolyzed as quercetin, it has been documented to cause alterations in the levels of testosterone and dihydrotestosterone (Becho *et al*., 2015). Chen *et al.* (2014) reported that higher doses of icariin, a flavonoid isolated from *Herba epimedii* plant, may damage reproductive functions by increasing oxidative stress in the testes. Saponins have also been reported to induce male reproductive toxicity, causing a decreases in sex organ weight, sperm count, sperm motility, and sperm density with histomorphological damage to the Sertoli and Leydig cells (Gupta *et al*., 2005). Therefore, the observed decrease in sex hormones and sperm parameters of MFMS-treated rats could be ascribed to the direct actions of the seed phytoconstituents on the testes.

Suffice to emphasize that HFMS had no effect on sex hormones and sperm profile. However, HFMS and MFMS significantly increased serum antioxidant levels (SOD and catalase) and significantly decreased lipid peroxidation, as evidenced by the decline observed in serum MDA. Catalase acts as a preventive antioxidant and SOD is a chain-breaking antioxidant enzyme that repairs cells and reduces the damage done to them by superoxides (Dinkova-Kotsova, 2002). The primary role of catalase is to scavenge hydrogen peroxide that has been generated by free radicals or by SOD in removal of superoxide anions and convert it to water. The two play key roles in the protection against the injurious effects of lipid peroxidation. Where SOD stops its function, catalase exerts its function (Petrulea *et al*., 2012). Due to oxidative stress, reactive oxygen species cause progressive damage to lipid macromolecules in a process called lipid peroxidation. Peroxidation of lipid membranes leads to loss of membrane fluidity and elasticity, impaired cell function, and even cell rupture. Malondialdehyde (MDA) is the terminal product of lipid peroxidation and serves as its index. This biomarker of oxidative stress was significantly decreased in the rats treated with *M. oleifera*. Lipid peroxidation can indirectly reflect the status of the metabolism of free radicals, the degree to which the cells are attacked by free radicals, and the degree to which lipid undergoes peroxidation (Petrulea *et al*., 2012). The increase in both SOD and catalase and the decrease in MDA observed in this study indicates that *M. oleifera* seeds are potentially capable of scavenging superoxides and reactive oxygen species, thereby decreasing lipid peroxidation and preventing free radical damage to cell membranes. HFMS had a greater effect on antioxidant levels than MFMS. Therefore, the bioactive agent in *Moringa oleifera* seeds responsible for the antioxidant properties and inhibition of lipid peroxidation generated *in vivo* is more concentrated in HFMS than in MFMS.

## CONCLUSION

In conclusion, the methanol fraction of *Moringa oleifera* seeds induced toxicity to male rat reproductive physiology by decreasing testicular androgen. However, the hexane fraction of *Moringa oleifera* seeds has no reproductive toxicity effect and was demonstrated to possess greater antioxidant potentials than the methanol fraction.
